# An In Situ Prepared Comb-like Polycaprolactone-Based Gel Electrolyte for High-Performance Lithium Metal Batteries

**DOI:** 10.3390/ma16052117

**Published:** 2023-03-06

**Authors:** Yange Fan, Huifeng Wang, Shipeng Chen, Yimin Hou, Shujiang Wang

**Affiliations:** 1 Institute of Chemistry Co., Ltd., Henan Academy of Sciences, Zhengzhou 450002, China; 2 School of Materials Science and Engineering, Zhengzhou University, Zhengzhou 450001, China

**Keywords:** gel electrolyte, in situ preparation, comb-like polycaprolactone, lithium metal batteries

## Abstract

Herein, we present the synthesis and electrochemical performance of a comb-like polycaprolactone-based gel electrolyte from acrylate terminated polycaprolactone oligomers and liquid electrolyte for high-voltage lithium metal batteries. The ionic conductivity of this gel electrolyte at room temperature was measured to be 8.8 × 10^−3^ S cm^−1^, which is an exceptionally high value that is more than sufficient for the stable cycling of solid-state lithium metal batteries. The Li^+^ transference number was detected to be 0.45, facilitating the prohibition of concentration gradients and polarization, thereby prohibiting lithium dendrite formation. In addition, the gel electrolyte exhibits high oxidation voltage up to 5.0 V vs. Li^+^/Li and perfect compatibility against metallic lithium electrodes. The superior electrochemical properties provide the LiFePO_4_-based solid-state lithium metal batteries with excellent cycling stability, displaying a high initial discharge capacity of 141 mAh g^−1^ and an extraordinary capacity retention exceeding 74% of its initial specific capacity after being cycled for 280 cycles at 0.5C at room temperature. This paper presents a simple and effective in situ preparation process yielding an excellent gel electrolyte for high-performance lithium metal battery applications.

## 1. Introduction

Secondary lithium-ion batteries (LIBs) are widely employed as a sustainable energy source for applications in energy conservation devices such as portable electronics, electric vehicles (EVs), etc. [1]. However, limitations, such as restricted energy density and safety risks seriously hinder the full realization of the potential of LIBs in emerging applications of EVs and power grids. To achieve higher energy density, the use of metallic lithium as anodes is favored, owning to its extraordinary theoretical capacity (3860 mAh g^−1^) and superior redox voltage (−3.04 V) [2]. Unfortunately, the widely used traditional organic liquid electrolytes (LEs) are limited because of their high flammability and high reactivity with metallic lithium, which result in hazardous incidents [3]. To address this challenge, the development of solid-state lithium metal batteries with solid-state electrolytes (SSE) instead of traditional LEs has emerged as an important strategy that offers improved energy and safety [4]. Given the key role of solid-state lithium batteries, polymer-based solid-state electrolytes are widely considered superior to inorganic solid-state electrolytes due to their favorable properties, including good processability, high elasticity, and perfect interfacial self-adjusting with electrodes [5,6].

Significant efforts have been invested in the development of advanced solid-state polymer electrolytes [7]. A wide range of polymer matrices have been explored for SPEs, including polyethylene oxide [8], polycarbonate [9,10], polyacrylonitrile [11], poly(vinylidene fluoride) [12], etc. Despite these efforts, solid-state polymer electrolytes are often characterized by insufficient ionic conductivity at ambient temperatures (<10^−5^ S cm^−1^) and high interfacial impedance [8,13], resulting in insufficient cycling stability and poor operational performance. Their inadequate ionic conductivity at room temperature is mainly attributed to the semicrystalline nature of these polymer main chains and restricted segmental motions [8,9,10]. Various strategies have been developed to enhance ionic conductivity, such as the incorporation of inorganic additives [14], blending [15], copolymerization [16], crosslinking [17], etc. However, low ionic conductivity at ambient temperatures remains a significant barrier to the practical application of solid-state polymer electrolytes. In addition, the uncontrolled growth of lithium dendrites represents a constraint to the practical application of lithium metal batteries [18]. Therefore, the development of novel high-performance, polymer-based, solid-state electrolytes that provide sufficient electrochemical properties for lithium batteries is a hot research area in the energy conservation field. 

As discussed above, to date, no solid-state polymer electrolyte has been able to satisfactorily fulfill the critical requirements for lithium metal batteries, particularly with regard to insufficient ionic conductivity at ambient temperatures. Nevertheless, polymer-based gel electrolytes (GEs) have garnered significant attention in recent years due to their remarkable attributes, including high ionic conductivity, improved interfacial contacts with the electrodes, sufficient mechanical strength, and their shape-formation capabilities [19]. These properties make GEs a highly promising solution to address safety concerns and inhibit the formation of lithium dendrites in lithium metal batteries. For instance, Yang et al. successfully synthesized polyether-based GEs through the polymerization of 1,3-dioxolane, which displays remarkable non-flammability and ionic conductivity (3.37 × 10^−4^ S cm^−1^) at room temperature [20]. In addition to demonstrating high compatibility with metallic lithium anodes, these GEs effectively suppresses the formation of lithium dendrites during lithium plating/stripping processes. Moreover, Kim et al. reported on the synthesis of highly conductive GEs by click chemistry consisting of an ionically conductive oligomer (polycaprolactone triacrylate) and an organic solvent [21]. These GEs exhibit high ionic conductivity (2.2 × 10^−3^ S cm^−1^) at room temperature, as well as excellent cycling performance, improved thermal stability, and no organic solvent leaking risk under high pressure. GEs offer a perfect balance between the physical and chemical properties of LEs and solid-state polymer electrolytes, as they constrain LEs within the polymer matrix, providing a potential compromise between safety and feasibility through precise regulation of the content of the LEs and the polymer matrix. 

Additionally, the application of in situ polymerization techniques has emerged as a highly promising approach for the preparation of GEs, as it enables the simultaneous synthesis of the polymer electrolyte and cell assembly. This obviates the laborious and environmentally detrimental procedures associated with traditional ex situ preparation and enhances the interconnection between the electrolyte and electrodes, thereby reducing contact resistance [22]. In a study reported by Duan et al., GEs were fabricated through in situ thermally initiated polymerization, which exhibited a lower interfacial resistance and elevated ionic conductivity (2.52 × 10^−4^ S cm^−1^ at ambient temperature) compared to the ex situ prepared cells [23]. Furthermore, Chen et al. synthesized an in situ GE composed of polyethylene glycol and LiPF_6_ in carbonate solvents, which manifested high electrochemical performances (3.35 × 10^−3^ S cm^−1^ at ambient temperature; high electrochemical stability of up to 4.9 V) [24]. Lithium dendrite formation during charge–discharge cycling is effectively mitigated through the enhancement of the solid electrolyte interphase (SEI) and the outstanding contact interface. 

Herein, a simple and facile approach is presented for the synthesis of a comb-like polycaprolactone-based GE from acrylate-functionalized polycaprolactone (PCL) through the utilization of an efficient thermally initiated radical polymerization technique. The chemical structure of the reactive PCL-based precursor was thoroughly confirmed by proton nuclear magnetic resonance spectroscopy (^1^H NMR) and Fourier transform infrared spectroscopy (FTIR). A comprehensive evaluation of the electrochemical properties was performed, including assessments of ionic conductivity, lithium-ion transference number, and compatibility with metallic lithium. Solid-state lithium metal batteries comprising LiFePO_4_/GE/Li were assembled and subjected to evaluations at different discharge rates. Given the high ionic conductivity and excellent interface between the GE and electrodes, the solid-state batteries exhibit extraordinary rate and cycling performances under room-temperature conditions. 

## 2. Experimental

### 2.1. Materials

The raw materials for the synthesis of acrylate-functionalized polycaprolactone oligomers, i.e., 1-butanol (99%), ε-caprolactone (99%), tetrahydrofuran (99%), triethylamine (97%), and toluene (99%), were all purchased from Aladdin (Shanghai, China) and dried over CaH_2_. Acryloyl chloride (96%) was obtained from Aladdin. 1,5,7-triazabicyclo[4.4.0]dec-5-ene (98%, TBD) was purchased from J&K Chemical. 2,2′-Azobis(2-methylpropionitrile) (99%, AIBN) was purchased from Aladdin and used after recrystallization in methanol. Lithium hexafluorophosphate (99.95%, LiPF_6_) from Sigma Aldrich (Waltham, MA, USA) was used as the lithium salt for GE. Cathode-active materials of LiFePO_4_, carbon black (Super P), metallic Li sheets, and Al foil were purchased from Shenzhen Kejing Star Technology Co., Ltd. (Shenzhen, China). The LE used in this work was composed of ethyl methyl carbonate (EMC) and ethylene carbonate (EC) in a ratio of 7:3 (*v*/*v*) with 1 M LiPF_6_.

### 2.2. Preparation of PCLA

**Scheme 1:**
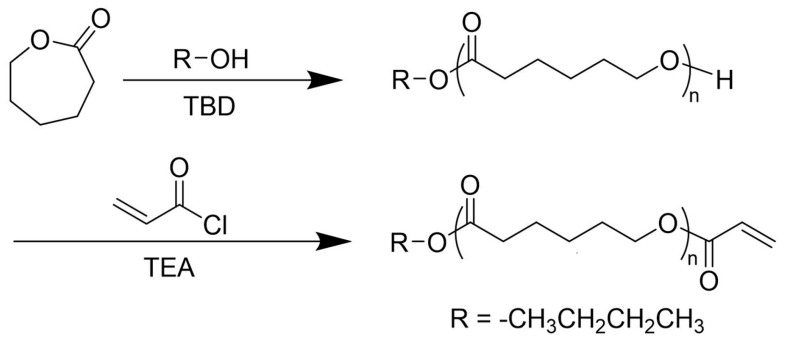
The synthesis of PCLA.

The synthesis of acrylate-functionalized PCL precursor (PCLA) was performed according to a previously reported literature procedure [14], as shown in Scheme 1. A typical synthetic process for PCLA us as follows: To a dry 250 mL flask, 45.66 g of ε-caprolactone (400 mmol), 6.67 g of 1-butanol (90 mmol), 0.28 g of TBD (2 mmol), and 100 mL of toluene were added. After degassing and refilling with Ar, the reaction solution was stirred by a magnetic stirring bar at room temperature for 24 h. After polymerization, the colorless oil was washed with brine 3 times to remove TBD, dried over MgSO_4_, and concentrated by a rotary evaporator. This reaction yielded a colorless oil, i.e., polycaprolactone oligomer, which was then dried at 60 °C under vacuum for 12 h, yielding 46.58 g.

The obtained polycaprolactone oligomer was reacted with 14.12 g of acryloyl chloride (156 mmol) in 80 mL tetrahydrofuran at 0 °C in an ice bath, using 15.78 g of triethyl amine (156 mmol) as the acid scavenger. The slightly yellowish mixture was then stirred at room temperature for another 20 h. After removing the precipitation of triethylamine hydrochloride, the oil was concentrated using a rotary evaporator. The obtained crude product was then dissolved in 300 mL dichloromethane, washed 3 times, and dried over anhydrous MgSO_4_. A slightly yellowish oil was obtained after removing the solvent and dried under vacuum at 30 °C for 24 h, yielding 49.80 g.

### 2.3. Fabrication of GE Membrane

The comb-like PCL-based GE was prepared via facile in situ radical polymerization inside an argon-filled glove box (H_2_O, O_2_ < 1 ppm). A mixture composed of 2 g of PCLA, 8 g of LE, and 0.02 g of AIBN was prepared and stirred for 10 min at room temperature in the glove box. The weight fraction of the polymer in the GE was tuned by varying the PCLA/LE ratios. The colorless, clear solution was then poured into a level Teflon Petri dish with a piece of cellulose nonwoven membrane inside. The Teflon Petri dish was sealed to prevent the evaporation of LE while curing at an elevated temperature. A solid, colorless membrane was obtained after curing at 80 °C inside the glove box for 6 h. The GE-based solid-state batteries were all prepared by injecting the PCLA/LE mixture into the cell, sealing the cell case, and curing at 80 °C for 6 h.

### 2.4. Characterization

The chemical composition of the PCLA was thoroughly studied by proton nuclear magnetic resonance spectroscopy (^1^H NMR, Bruker Ascend TM 400 MHz) with CDCl_3_ as the solvent and the tetramethylsilane (TMS) as the internal standard. Fourier transform infrared spectroscopy (FTIR) measurement of the PCLA was performed before and after curing on a Nicolet^TM^ iS50 FTIR spectrometer in the wavenumber range of 4000 to 600 cm^−1^. Microscale observation of the GE film and the lithium electrode surface was conducted using a TESCAN MIRA3 field emission scanning electron microscope.

The electrochemical properties of the GE were all characterized on a Solartron 1260 multichannel potentiostat electrochemical workstation. Electrochemical impedance spectroscopy (EIS) with an AC amplitude of 10 mV was applied to measure the ionic conductivities of the GEs with various LE concentrations in the frequency range of 10^6^ to 10^−2^ Hz. In this case, symmetrical SS//SS structured cells with GE sandwiched between stainless-steel sheets (SS) were assembled. The ionic conductivity (σ, S cm^−1^) was evaluated using Equation (1):

(1) $$\sigma =\frac{L}{R_{b}A}$$

where *L* is the thickness of the GE (in cm), *R_b_* is the impedance of the GE (in Ω), and *A* is the overlapping area of the GE and SS (in cm^2^).

The oxidation stability of the GE was evaluated using linear sweep voltammograms at 25 °C between 0 V and 7 V vs. Li/Li^+^ at a scanning rate of 1.0 mV s^−1^. In this case, a stainless-steel sheet was employed as the working electrode, while a metallic lithium sheet was used as the counter and reference electrode.

To measure the lithium-ion transference number (*t_Li_^+^*) of the GE, a Li/GE/Li symmetrical cell was assembled, and the impedance was tested before and after polarization at room temperature. The *t_Li_*^+^ value was calculated using Equation (2):

(2) $${t_{Li}}^{+}=\frac{I_{ss}\left(\Delta V-I_{0}R_{0}\right)}{I_{0}\left(\Delta V-I_{ss}R_{ss}\right)}$$

where *I*_0_ and *I_ss_* denote the initial and stable currents, respectively, and *R*_0_ and *R_ss_* denote the interfacial impedance between the GE and the metallic lithium electrodes prior to and following polarization, respectively. The applied DC polarization voltage was 10 mV (Δ*V*).

The lithium dendrite prohibition property of the GE was evaluated via a galvanostatic cycling study on symmetrical cells of Li/GE/Li on a Shenzhen Neware battery testing system (charged/discharged both for 1 h at 0.1 mA cm^−2^).

The long-term cycling and rate performances of the batteries were tested using a Shenzhen Neware battery testing system. The C-rate was determined according to 1C = 170 mAh g^−1^, and the applied voltage range was between 2.8 and 3.8 V. A commercial high-loading LiFePO_4_ (LFP) cathode was used in our work, with an loading area of ~15 mg cm^−2^. All the rate and cycling performance tests were conducted by assembling the LFP/GE/Li structured CR-2032 coin cells.

## 3. Results and Discussion

### 3.1. Chemical Characterization

**Figure 1:**
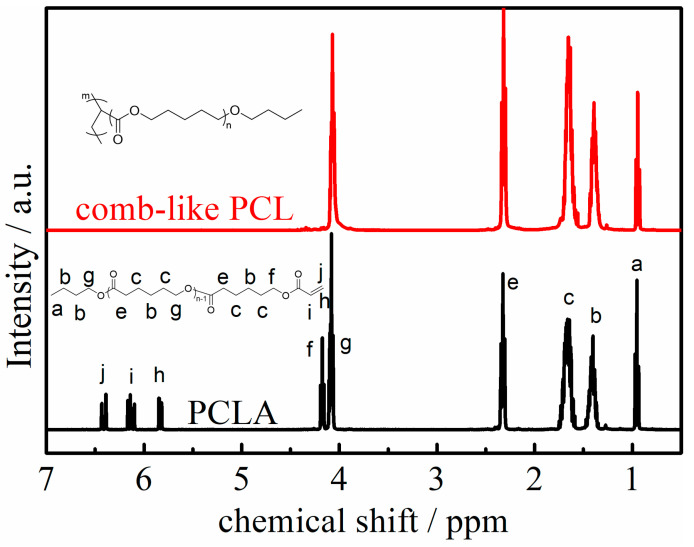
The ^1^H NMR results of the PCLA precursor and comb-like PCL in CDCl_3_.

The synthesis of the PCLA precursor was carried out by preparing the PCL oligomer by cationic ring-opening chemistry of ε-caprolactone using a strong base, followed by modification of the hydroxy end groups with acryloyl chloride towards PCLA. PCLA was synthesized according to a previously described procedure, as shown in Scheme 1 [14]. The received PCLA precursor was confirmed by an ^1^H NMR study, and the proton assignments are depicted in Figure 1.

**Figure 2:**
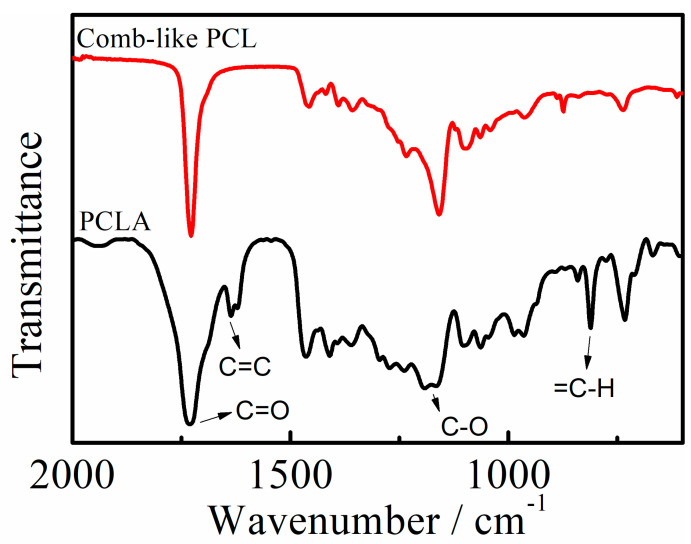
The FTIR results of the PCLA precursor and the comb-like PCL thereof.

As shown in Figure 1, the proton signals of a (chemical shift, δ = ~0.95 ppm), b (δ = ~1.4 ppm), and g (δ = ~4.1 ppm) are attributed to the 1-butyl end groups from the 1-butanol initiator. The proton signals of c (δ = ~1.7 ppm), e (δ = ~2.3 ppm), and g (δ = ~4.1 ppm) are assigned to -C*H*_2_-, -CO-C*H*_2_-, and –C*H*_2_-O- on the PCLA backbone, respectively. The proton signal of f (δ = ~4.2 ppm) is due to the –C*H*_2_-O- in the acrylate end functionalities. The proton signals at 5.8 ppm (h), 6.1 ppm (i), and 6.4 ppm (j) are all assigned to the double bonds of the acrylate functionalities. The proton signal assignments confirm the successful synthesis of acrylate-terminated PCLA precursors. According to integration of the peak area of the end groups and the backbone characteristic signals, the average molecular weight (Mn) of the PCLA precursor is ~650 g·mol^−1^, which is very close to the theorical value. The successful preparation of PCLA precursor is also reflected by the FTIR measurements, as shown in Figure 2.

The bands at 1760 cm^−1^ and 1106 cm^−1^ are assigned to the C=O stretching vibrations and the C-O symmetric stretching vibration, respectively. The characteristic stretching vibration of C=C is located at 1638 cm^−1^, and the deformation vibration of =C-H was detected at 1410 and 810 cm^−1^.

After curing the PCLA and LE mixture into a GE, the chemical structure of the comb-like PCL was confirmed by ^1^H NMR and FTIR, as shown in Figure 1 and Figure 2, respectively. In the ^1^H NMR spectra of comb-like PCL, the proton signals at 5.8 ppm (h), 6.1 ppm (I), and 6.4 ppm (j) assigned to the double bonds of the acrylate functionalities of PCLA all disappear, indicating the full reaction of the double bonds. This was further confirmed by the disappearance of the proton signal of f (δ = ~4.2 ppm) due to the –C*H*_2_-O- in the end groups. All other proton signals attributed to the backbone of the PCL oligomer were retained. In the FTIR spectrum of comb-like PCL, the characteristic stretching vibration of C=C located at 1638 cm^−1^ totally disappeared, confirming the end-group modification reaction. The FTIR results are in line with the results of the ^1^H NMR experiment, both confirming the successful thermally initiated polymerization of PCLA.

### 3.2. Ionic Conductivity

**Figure 3:**
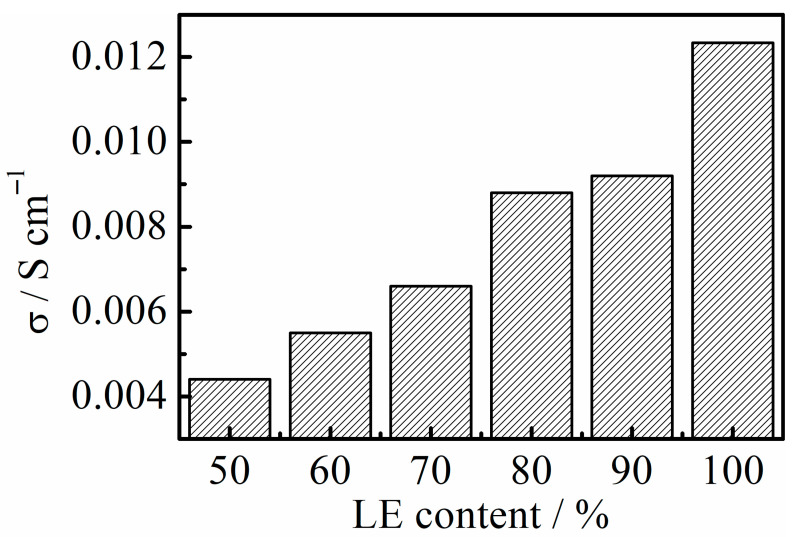
The ionic conductivity of the GE vs. LE contents at 25 °C.

Sufficient ionic conductivity is the basis for the stable operation of solid-state lithium metal batteries. The ionic conductivity (σ) of the comb-like PCL-based GE as a function of LE contents at 25 °C is depicted in Figure 3.

In general, ionic conductivity is enhanced with LE contents, reaching 8.8 × 10^−3^ S cm^−1^ at an LE content of 80%. A further increase in the LE proportions does not result in a significant increase in ionic conductivity. This is clearly due to the fact that the ionic conduction of the LE governs the overall ion transference, even though the comb-like PCL may have some strong interactions with the cations and anions. It should be noted that the ionic conductivity measured in this work was significantly higher than that of other GEs, such as the poly(ether-ester)-based GE reported by Lei et al. [25]. Because the ionic conductivity plateau was first achieved at an LE content of 80%, the GE with an LE content of 80% was chosen for subsequent experiments.

### 3.3. Morphology of the GE

**Figure 4:**
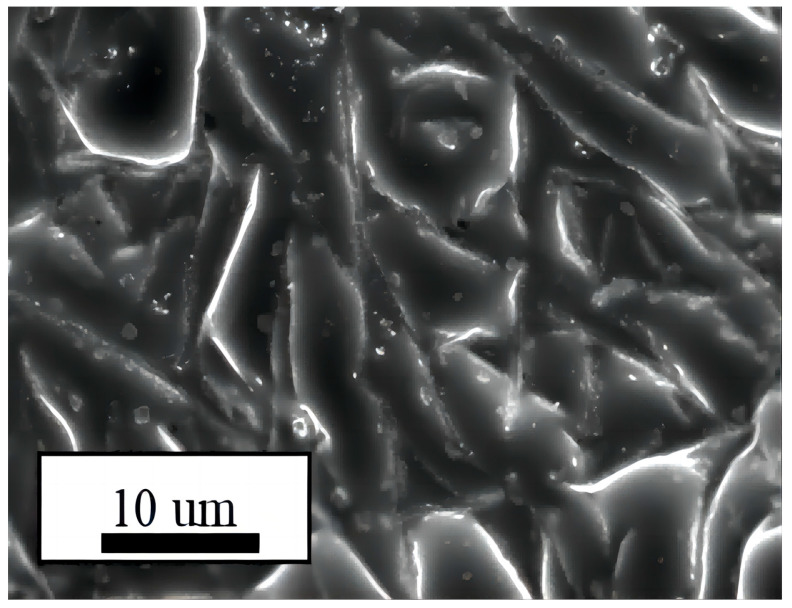
SEM image of the cured comb-like PCL-based GE membrane.

After the formation of the GE, the facial morphology of the comb-like PCL-based GE membrane with an LE content of 80% was analyzed by scanning electron microscopy (SEM, Figure 4).

No obvious phase separation was observed, indicating the homogeneous nature of the comb-like PCL-based GE. The SEM image also confirms the complete solvation of LiPF6 in the GE polymer matrix, with no signs of detectable insoluble lithium salt particles. The comb-like PCL-based GE membrane exhibits no obvious crystalline structure at the microscopic level. The homogeneous nature and high solubility of the lithium salt in the GE highly facilitates the fast transition of lithium ions.

### 3.4. Electrochemical Stability of the GE

**Figure 5:**
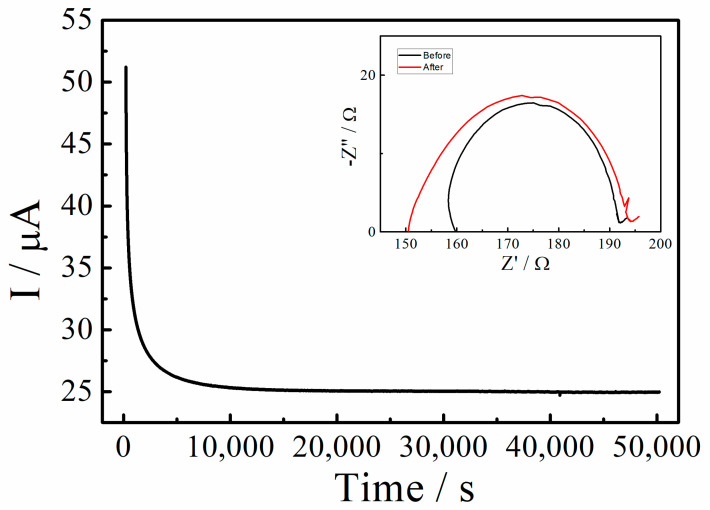
Chronoamperometry of the Li/GE/Li symmetrical cell at 25 ℃. The inset shows the AC impedance spectra before and after polarization.

The lithium-ion transference number (*t_Li_^+^*) of a GE is an essential basis for the battery cycling performance. An ideally high lithium-ion transference number would be close to unity, as it plays an important role in preventing the Li^+^ concentration gradients during battery operation. These Li^+^ concentration gradients are thought to be the cause of various battery defects, including uncontrollable lithium dendrites and charging rate restrictions [26]. In our work, the methodology developed by Evans and Vicent was employed to measure the lithium-ion transference number [27]. The EIS and polarization test results of GE-based Li//Li symmetrical cells are depicted in Figure 5.

Calculation according to Equation (2) shows that the *t_Li_^+^* value of the comb-like PCL-based GE is as high as 0.45. In sharp contrast, the *t_Li_^+^* value of the LE was measured to be only 0.2, which is significantly lower than that of the GE. This is most likely due to the fact that the promotion effect of the polymer matrix on Li^+^ ion conduction. In addition, the *t_Li_^+^* of the GE is also much higher than that of classical polyethylene oxide-based solid-state polymer electrolytes (<0.2) [28,29]. Given that the carbonyl groups in the polymer matrix interact with the Li^+^ ions less strongly than polyethylene oxide, the comb-like PCL matrix enables Li^+^ transport via a different mechanism [9].

**Figure 6:**
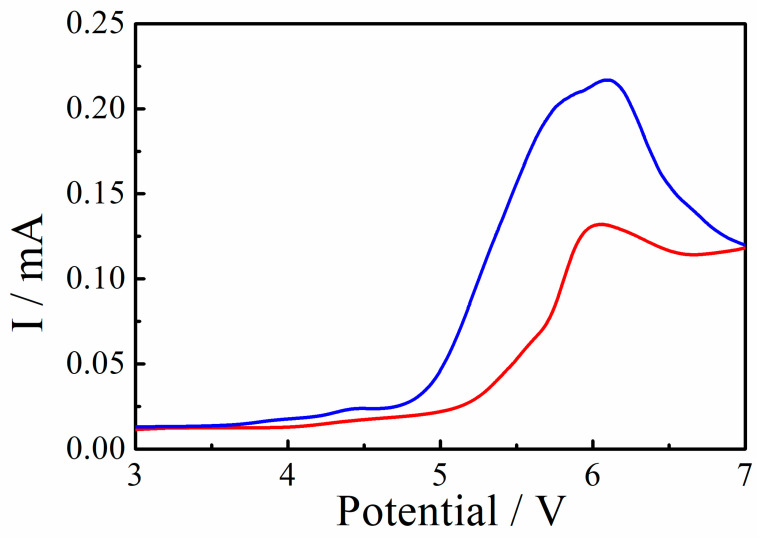
The linear voltammetry curves of the comb-like PCL-based GE (red) and traditional LE (bule) at 25 °C.

Figure 6 depicts the electrochemical stability results of the comb-like GE as evaluated by linear sweep voltammetry. The in situ polymerized comb-like PCL-based GE exhibits no oxidative decomposition before 5 V in its LSV curve. In sharp contrast, the electrochemical stability of the LE is marginally compromised before 4.6 V. The significant difference in the electrochemical stability of the LE and GE is believed to be derived from the enhancing effect of the polymer matrix. The comb-like polycaprolactone has a polyester skeleton, which has a low HOMO level, thereby enabling a GE with an obviously higher electrochemical stability window. The widely utilized lithium iron phosphate-based cathode (LiFePO_4_, LFP) has a low electrochemical stability window requirement (working voltage of 3.5 V vs. Li/Li^+^) that can be satisfied by the current comb-like PCL-based GE [30].

### 3.5. Interfacial Stability against Lithium Metal Electrode

**Figure 7:**
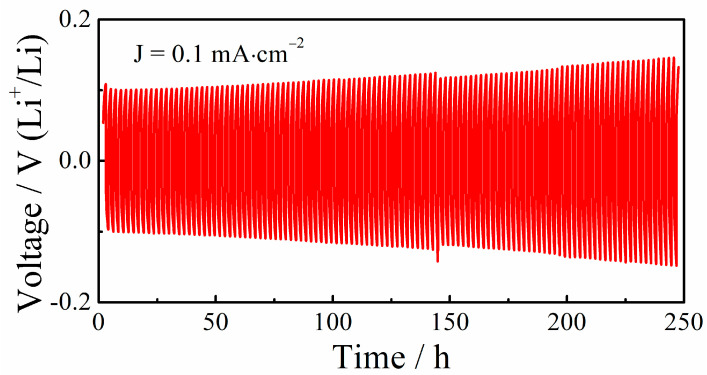
Chronopotentiometry results obtained in the Li plating/stripping test (0.1 mA cm^−2^, 0.1 mAh cm^−2^).

The operation performance of solid-state lithium metal batteries is greatly influenced by the interfacial stability between Li and electrolytes [31]. Chronopotentiometry results obtained in the Li plating/stripping test are shown in Figure 7, with a current density of 0.1 mA cm^−2^ and a whole plating/stripping cycle period of 2 h at room temperature. A negative voltage on this curve represents the Li^+^ plating, whereas a positive voltage represents the Li^+^ stripping. The symmetrical cell using the comb-like PCL-based GE cycles continuously and stably for more than 250 h without experiencing any noticeable negative effects or short circuits. The outstanding performance is most likely due to the excellent adhesive qualities of the in situ polymerized comb-like PCL-based GE, which improved the interfacial contact between the metallic lithium electrodes and the electrolyte membranes [32,33,34]. In addition, the GE exhibits high *t_Li_^+^*, which decreases the concentration gradients and polarization [16]. Consequently, lithium dendrite is prohibited. In sharp contrast, the Li//Li symmetrical cells in PEO-based solid-state polymer electrolyte systems [30] suffer from subpar cycling performances, leading to rapid failure. The in situ polymerized comb-like PCL-based GE investigated in this work significantly improves the interfacial stability of the Li electrode with electrolytes.

### 3.6. Cell Performance

The rate capacity and cycling performances of the solid-state lithium metal batteries based on the comb-like PCL-based GE were evaluated by assembling LFP/GE/Li cells. To achieve perfect interfacial contact between the GE and cathode, a comb-like PCL-based GE film was fabricated by in situ curing in sealed CR-2032 cells under thermal initiation. Adequate interfacial contact between the GE membranes and the cathodic active materials guarantees the full operational potential of solid-state battery systems [35]. In this study, LiFePO_4_ was selected as the cathodic active material due to its superior stability in comparison to other alternatives [36].

In general, the transport efficiency of Li^+^ by the solid-state electrolyte itself and the interface between the electrolyte and electrodes play a critical role in determining the rate performance of solid-state batteries.

**Figure 8:**
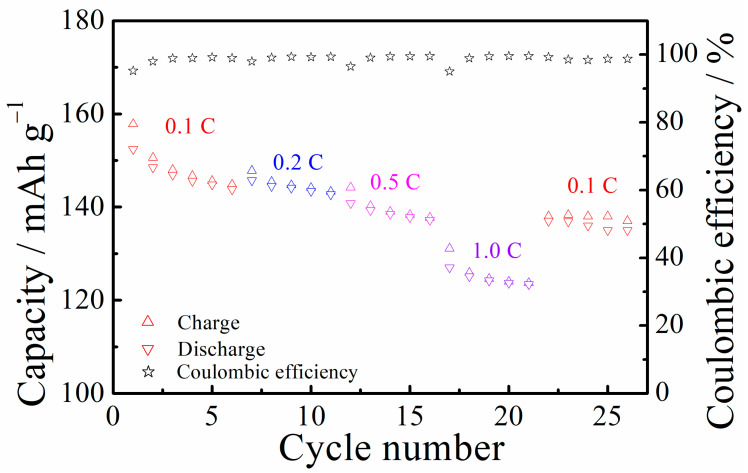
The rate performance of the LFP/GE/Li solid-state battery.

Figure 8 and Figure S1 show the discharge rate capacity of the LFP/GE/Li solid-state lithium metal batteries, which is based on the in situ cured comb-like PCL-based GE at room temperature and within a voltage range of 2.8 to 3.8 V and a C-rate range of 0.1 to 1C. At a C rate of 0.1C, the cell displays a specific capacity of 149 mAh g^−1^, which is approximately 93% of the theoretical capacity (160 mAh g^−1^) of LiFePO_4_ [37]. When the charge/discharge current increased to 0.2C, 0.5C, and 1C, the cell displayed reversible discharge capacities of 145 mAh g^−1^, 139 mAh g^−1^, and 125 mAh g^−1^, respectively, which are roughly 91%, 87%, and 78% of the theoretical discharge capacity values, respectively. Throughout testing, the solid-state cell exhibited a Coulombic efficiency of approximately 99%. These experimental results are very promising for solid-state batteries, as they demonstrate a capacity near the theoretical limit of LiFePO_4_ at high current rates. This is a significant improvement over the performance of PEO-based solid-state electrolytes, suggesting that the current comb-like PCL-based GE exhibits strong ionic conduction and excellent interface characteristics [38,39]. Finally, when the charge/discharge current was reduced from 1C to 0.1C, the discharge-specific capacity returned to 138 mAh g^−1^, which is ~93% of its original capacity, indicating strong structural and electrochemical stability.

**Figure 9:**
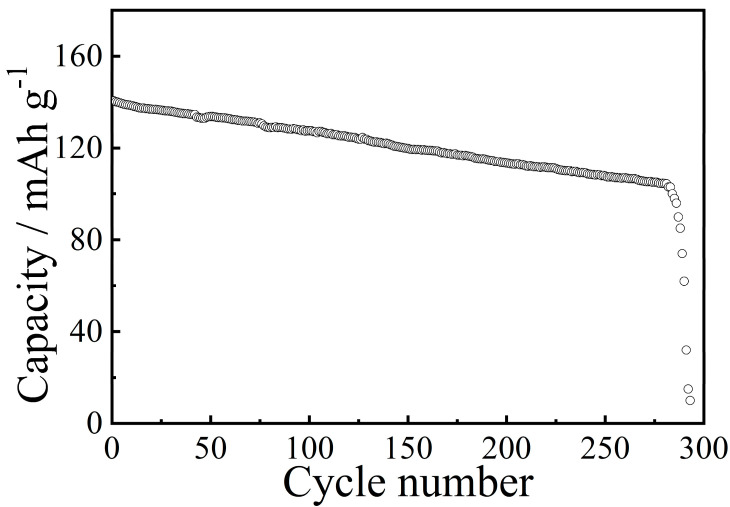
The long-term cycling performance of the GE-based LFP/GE/Li solid-state cell at 0.5C and 25 °C.

The discharge capacity retention profile of the LFP/GE/Li solid-state lithium metal battery is presented as a function of the cycle number at a C rate of 0.5C in Figure 9 and Figure S2. Basically, the solid-sate cell demonstrates remarkable cycling stability at room temperature, with a high initial discharge capacity (~141 mAh g^−1^) [40]. After being cycled for 280 cycles, the discharge capacity retention was more than 74% of its initial capacity, with a discharge capacity value of 105 mAh g^−1^. This excellent performance is attributed to the strong ionic conduction of the GE and effective interfacial contact between the electrodes and the GE generated by the in situ polymerization procedure. Compared with other solid polymer electrolyte-based lithium metal batteries, our GE can be cycled much more stably [34]. However, when the C rate was further increased to 1C, the solid-state battery did not perform very well. This decrease is probably a result of the higher concentration gradient polarization brought about by a high C rate and the limited charge carrier transference kinetics [41].

## 4. Conclusions

In conclusion, a new high-performance, comb-like, PCL-based GE was prepared with an acrylate-functionalized PCL precursor and a traditional liquid electrolyte via in situ thermally initiated radical polymerization. The chemical composition and structure of the reactive precursor of the PCLA were confirmed by ^1^H NMR and FTIR. SEM observation of the free-standing comb-like PCL-based GE membrane indicated a smooth, homogeneous surface. The GE exhibits a plateau in ionic conductivity (8.8 × 10^−3^ S cm^−1^) with LE content higher than 80%. Compared to the low lithium transference number of ~0.2, the GE shows a high *t_Li_*^+^ of approximately 0.45 due to the improvement effect of the PCL matrix. In addition, the GE displays high electrochemical stability up to 5.0 V vs. Li/Li^+^. The LiFePO_4_/GE/Li cells show extraordinary rate and cycling performance at room temperature, with a superior discharge capacity of 141 mAh g^−1^ and a high capacity retention rate of more than 74% after being cycled for over 280 cycles at a high C rate of 0.5C. The current in situ prepared comb-like PCL-based GE is a promising electrolyte candidate for high-energy, solid-state lithium metal batteries with excellent safety.

## Data Availability

The data could be provided if it is required by others.

## Supplementary Materials

The following supporting information can be downloaded at: https://www.mdpi.com/article/10.3390/ma16052117/s1.
