# Supplementary material for: An In Situ Prepared Comb-like Polycaprolactone-Based Gel Electrolyte for High-Performance Lithium Metal Batteries
Source: Materials (Basel). 2023 Mar 6;16(5):2117. doi: 10.3390/ma16052117 (PMC10003875; doi:10.3390/ma16052117)
Supplement: Supplementary file 1 [file materials-16-02117-s001.zip › materials-2233917-supplementary.pdf]

Electronic Supplementary Information for

An In Situ Prepared Comb-Like Polycaprolactone-based Gel Electrolyte for High-Performance Lithium Metal Batteries

Yange Fan [1,2,*], Huifeng Wang [1], Shipeng Chen [1], Yimin Hou [1] and Shujiang Wang [1]

[1] Institute of Chemistry Co. Ltd., Henan Academy of Sciences, Zhengzhou 450002, China

[2] School of Materials Science and Engineering, Zhengzhou University, Zhengzhou 45001, China

[*] Correspondence: fanyange@gs.zzu.edu.cn

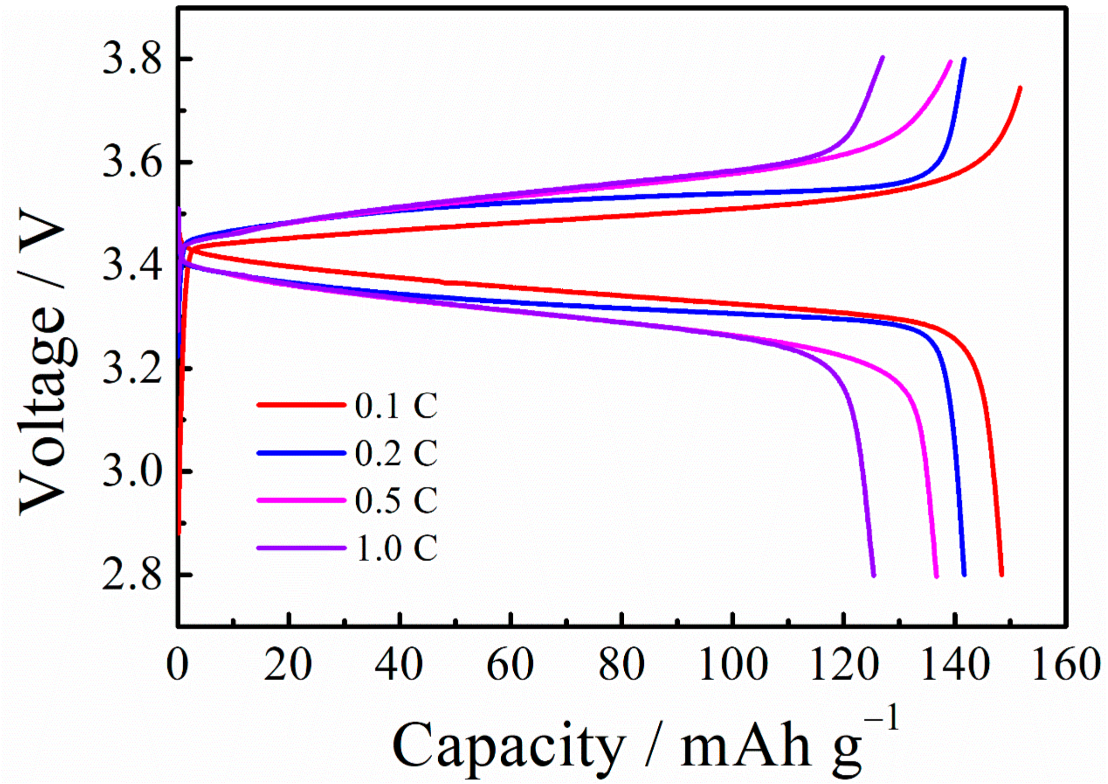

Figure S1. The charge/discharge profiles of the solid-state battery at various C-rates.

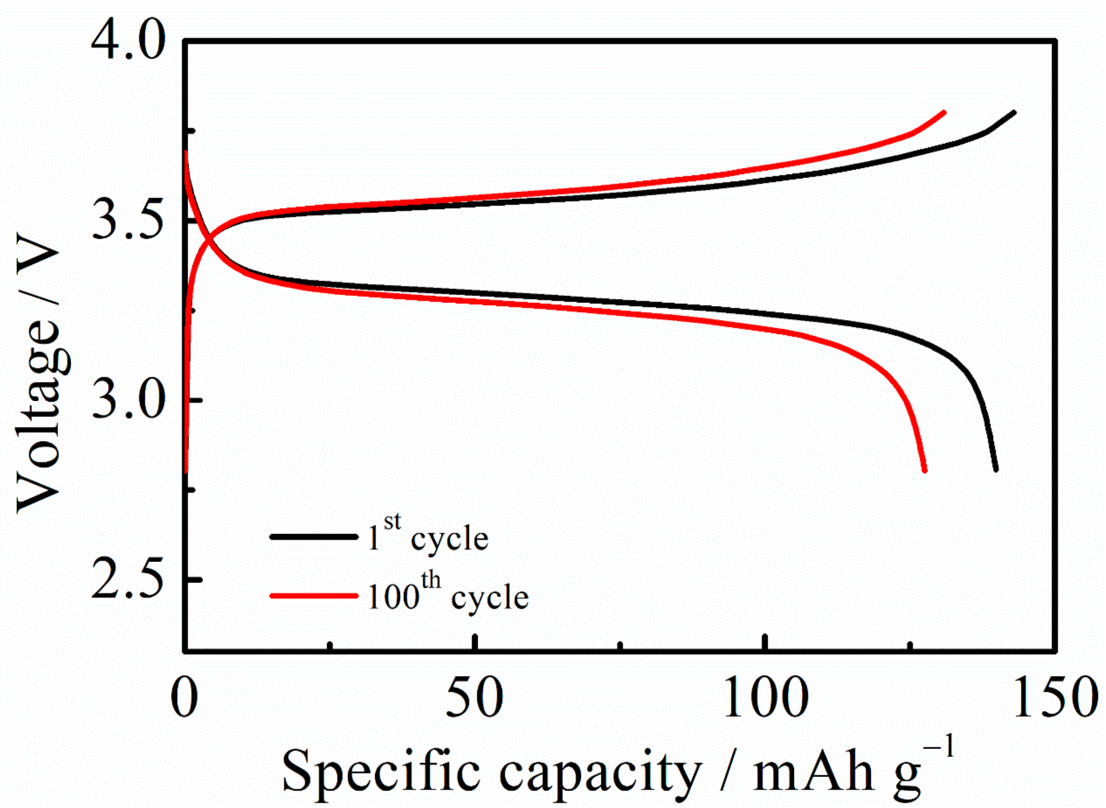

Figure S2. The charge/discharge profiles of the solid-state battery at 0.5C at 1st and 100th cycles.
